# *Delfinoia*, a new South American aphid genus (Hemiptera, Aphididae, Macrosiphini) on *Cayaponia* (Cucurbitaceae)

**DOI:** 10.3897/zookeys.671.12247

**Published:** 2017-04-26

**Authors:** Juan M. Nieto Nafría, M. Pilar Mier Durante, Sara I. López Ciruelos

**Affiliations:** 1 Departamento de Biodiversidad y Gestión Ambiental; Universidad de León; 24071 Leon

**Keywords:** Aphididae, aphids, Argentina, *Delfinoia*, Macrosiphini, new genus, Peru, species synonymy

## Abstract

The genus *Delfinoia* Nieto Nafría & Mier Durante **gen. n.** is established, and *Utamphorophora peruviana* (Essig), originally *Amphorophora peruviana* and currently *Delfinoia peruviana* **comb. n.**, is designated species type of the genus. The synonymy between this species and *Wahlgreniella australis* Delfino **syn. n.** is established. Apterous and alate viviparous females of *D. peruviana* are redescribed; the male is also described. The species is currently known from Peru and Argentina; a plant of the genus *Cayaponia* (Cucurbitaceae) is the only identified host.

## Introduction

*Utamphorophora peruviana* (Essig, 1953) and *Wahlgreniella australis* Delfino, 1981 are two South American macrosiphine aphids (Hemiptera, Aphididae, Macrosiphini) that have never been recorded after their respective descriptions.

**Figure 1.**
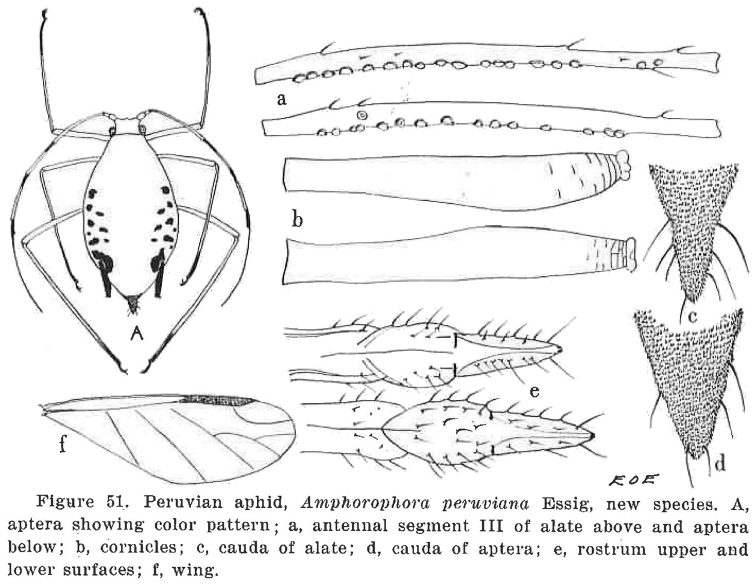
Illustration by Essig (“E O E”), with its legend, of the description of *Amphorophora peruviana*, on page 133 of his article (Essig, 1953). (Note: contrary to the legend, the antennal segment III attributed to an apterous viviparous female must be from an alate female, as Essig described the aptera (on page 135) as being without secondary sensoria).

*Utamphorophora peruviana* was described by Essig (1953) as *Amphorophora peruviana* from three alate and four apterous viviparous females, although he wrote three alatae and five apterae, which were “obtained by beating onto a canvas sheet” in Rio Pampas (Peru). This capture procedure allows us to speculate whether the host plant was a tree, or perhaps a shrub, but it also could be a vine climbing on a tree. The species was subsequently transferred to *Utamphorophora* Knowlton, 1946 by Eastop (in Remaudière and Remaudière 1997) without any explanation. Favret (2016) maintains this taxonomic position, which is nevertheless controversial because the ultimate rostral segment of the viviparous females of this species carries many accessory setae, as Essig (1953) illustrated (Fig. 1), while it has only two accessory setae in the viviparous females of the other currently known *Utamphorophora* species. The species is not included in the identification keys by Blackman and Eastop (2016) because its host plant was unknown.

*Wahlgreniella australis* was described (Delfino, 1981) from 11 alate and 16 apterous viviparous females collected from *Cayaponia* sp. in Cordoba (Argentina). *Cayaponia* (Cucurbitaceae) includes nearly 60 species, which characteristically are vine plants, and are spread over diverse territories of America from Oklahoma (USA) to Uruguay; several species have been recorded from Argentina, and three from Cordoba province, (Duchen and Renner 2010; Pozner 2016). The novelty of the aphid species and implicitly its generic adscription had been endorsed by D. Hille Ris Lambers (see Delfino 1981: 185). The species was maintained in *Wahlgreniella* Hille Ris Lambers, 1949 by Remaudière and Remaudière (1997), Blackman and Eastop (2016) and Favret (2016). Nevertheless this generic adscription is also debatable because the species exhibits some morphological characteristics that are different to those in other *Wahlgreniella* species; for example the triangular (rather than digitiform) cauda and the relatively weakly developed frontolateral tubercles. In addition all other *Wahlgreniella* species are North American or European in origin, and their host plants are species of *Rosa* and of Ericaceae in migrant species, or species of either *Rosa* or Ericaceae in monoecious species (Blackman and Eastop, 2016).

**Figure 2.**
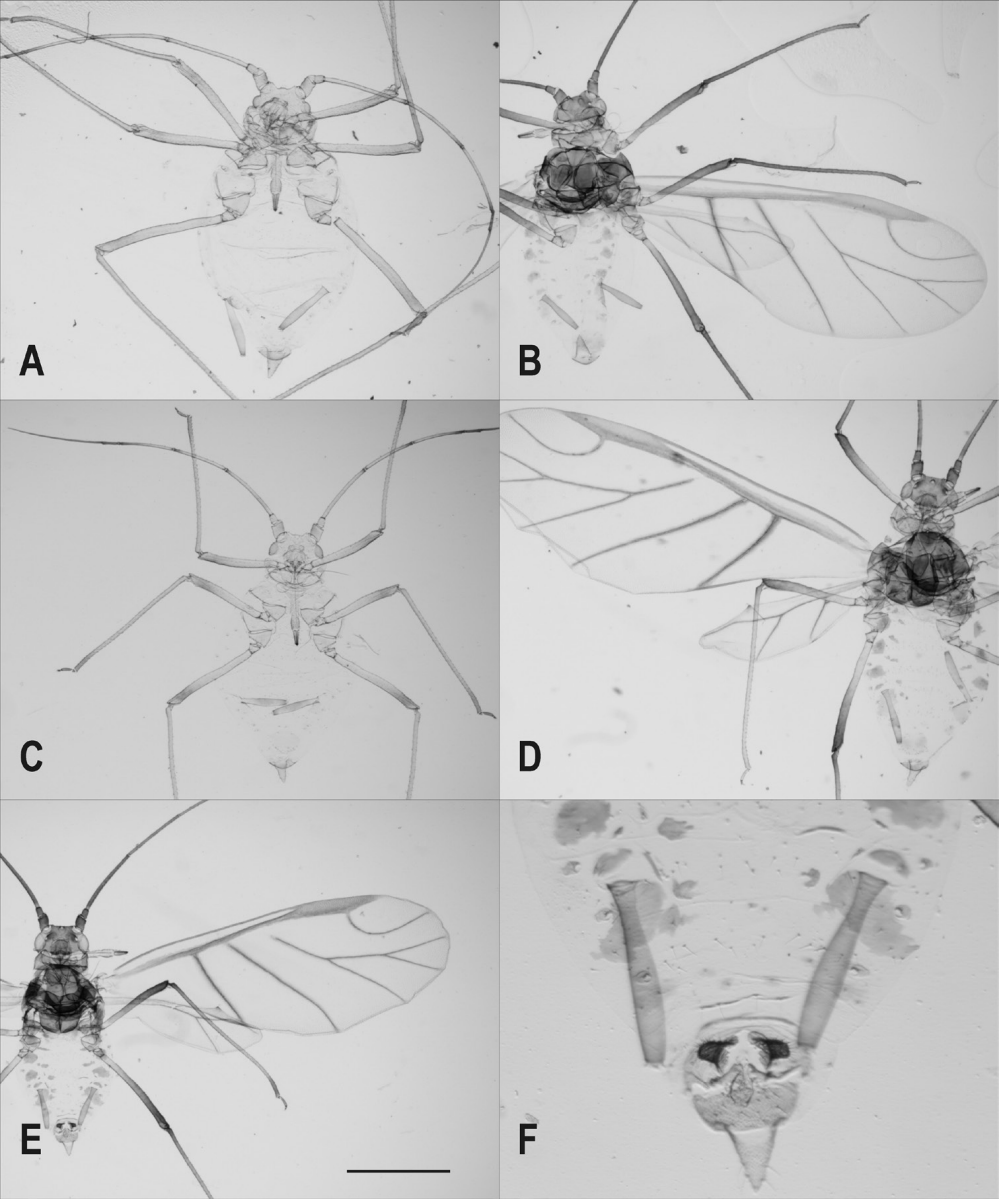
*Delfinoia peruviana* (Essig). **A–B** specimens from Peru, previously labelled *Utamphorophora peruviana* (Essig) **C–F** specimens from Argentina, previously labelled *Wahlgreniella australis* Delfino **A, C** apterous viviparous females **B, D** alate viviparous females **E–F** male. Scale bar: **A–D** 1 mm; **E, F** 0.21 mm.

Comparing the descriptions of the two species, and Essig’s drawings of *U. peruviana* (Fig. 1) with the prepared specimens of *W. australis* conserved in the collection of the *Muséum national d’Histoire naturelle* in Paris (France) (Fig. 2C, D), some similarities between them appear: shape of frons (divergent frontolateral tubercles present), length of antennal segment VI terminal process (near six times antennal segment VI base), shape of siphunculi (swollen, with long pedunculate proximal portion), cuticular ornamentation of siphunculi (absent in swollen portion), shape of cauda (triangular), setae of penultimate and ultimate rostral segments (abundant), secondary sensoria (only present on antennal segment III of alate viviparous females), and wing veins (cubitals dusky bordered).

The aim of this work is to contribute to knowledge of South American native aphid species by (1) increasing the known data of *Utamphorophora peruviana* from the re-examination of its types, (2) increasing the known data for viviparous females of *Wahlgreniella australis* and describing its male, and (3) reassessing the taxonomic position of these two nominal species.

## Materials and methods

Studied specimens of *Utamphorophora peruviana* (Essig): PERU: Rio Pampas (PERU) [possibly near Ayacucho, from the data base of the Essig Museum, University of California in Berkeley], March 8, 1951, beating [onto a canvas sheet], A. E. Michelbacher *leg.*; E. O. Essig *det.* (V-1951); three alate viviparous females and four apterous viviparous females; one alata, the holotype, in the California Academy of Science Entomological collection; others specimens, paratypes, in the Essig Museum of Entomology collection, University of California Berkeley.

Studied specimens of *Wahlgreniella australis* Delfino: (1) ARGENTINA, Cordoba [province], Cordoba [city], 16 May 1982, on *Cayaponia* sp., Delfino *leg.* & *det.*; 15 apterous viviparous females, six alate viviparous females and one male (winged); (2) ARGENTINA, Cordoba [province], Cordoba [city], barrio Cerro de las Rosas, 1 March1985, on an unidentified species of Brassicaceae, Bahamondes *leg.*, Remaudière *det.*; 11 apterous and three alate viviparous females. These are all located in the Muséum national d’Histoire naturelle (Paris, France) and Universidad de León aphidological collections.

**Figure 3.**
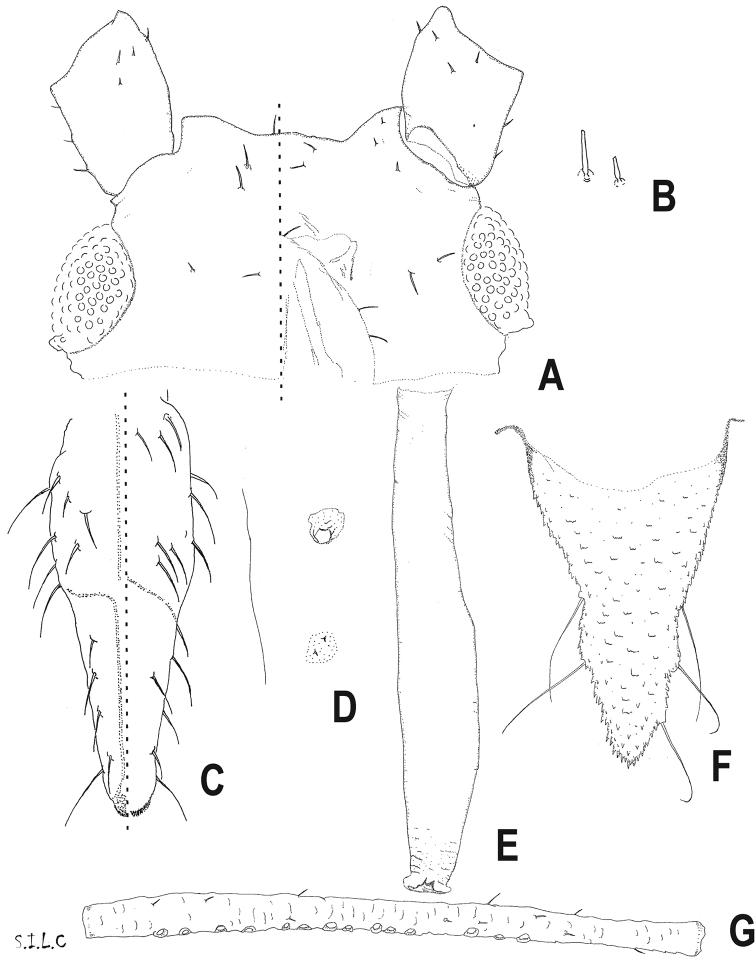
*Delfinoia peruviana* (Essig). **A–F** apterous viviparous females **G** alate viviparous female **A** frontal edge **B** dorsocephalic setae, third row, the shorter is the external one **C** pre-ultimate and ultimate rostral segments **D** spiracular abdominal plate and aperture **E** siphunculus **F** cauda **G** antennal segment III.

**Figure 4.**
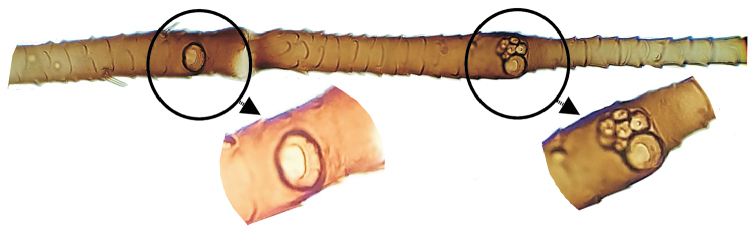
*Delfinoia peruviana* (Essig) apterous viviparous female. Antennal segments V and VI, in part, showing primary and satellite (on VI) sensoria.

Measurements were taken according to Nieto Nafría and Mier Durante (1998) with an ocular micrometer mounted on a light-field microscope. Microphotographs were taken with a Nikon set: SMZ1500 stereoscopic microscope with oblique coherent light, DXM1200F digital camera, and NIS-Elements F version 3.22 software (for Fig. 2), and with a smartphone through the eyepiece of a microscope Olympus CX41 (for Fig. 4). Drawings (Fig. 3) were made with the help of a camera lucida attached to the microscope.

## Results and discussions

The qualitative features of the studied viviparous females of *U. peruviana* (Essig) and *W. australis* Delfino are identical (Fig. 2) and the metric and meristic features are very similar (Table 1). Consequently, we propose that *Wahlgreniella australis* Delfino, 1981 syn. n. is a junior synonym of *Utamphorophora peruviana* (Essig, 1953).

**Table 1.** Metric and meristic features of viviparous females of *Utamphorophora peruviana* (Essig), and viviparous females and males of *Wahlgreniella australis* Delfino (all of them now under the name *Delfinoia peruviana*). The measurements are lengths, except where indicated that they are a width or diameter. Values in brackets are data from Delfino’s description of *W. australis*. Values in bold are new minima or maxima for each character in apterous or alate females based on our data. Abbreviations: AL, alate viviparous females; AP, apterous viviparous females; M, males; n, number of measured specimens; Abd., abdominal segment; AntIII, AntIV, AntV, AntVIb, AntVIpt, antennal segments, b and pt respectively being base and processus terminalis of sixth segment; *D*, subarticular width of AntIII; seg., segment; *SPW* and *SSW*, respectively minimal width of proximal pedunculate portion and maximal width of swollen portion of siphunculus.

	*U. peruviana* AP types (n=4)	*W. australis* AP new data (n=18) & [orig.descr.]	*U. peruviana* AL types (n=3)	*W. australis* AL new data (n=8) & [orig.descr.]	M n=1
Body [mm]	2.800–**3.575**	**2.150**–3.150	2.600–**3.125**	**2.350**–3.025	2.200
Antenna [mm]	3.875–**4.050**	**2.875**–**4.050**	3.600–**4.175**	**3.200**–3.875	3.513–3.675
Antenna / body [times]	**1.12**–1.42	1.18–**1.48**	1.31–1.39	**1.28**–**1.48**	1.60–1.67
AntIII [mm]	0.64–**0.90**	**0.58**–0.79	0.71–**0.88**	**0.63**–0.72	0.66–0.67
AntIV [mm]	**0.45**–0.78	0.50–**0.80**	0.65–**0.80**	**0.53**–0.69	0.60–0.62
AntV [mm]	**0.38**–0.71	0.51–**0.77**	0.59–**0.75**	**0.56**–0.69	0.60–0.61
AntVIb [mm]	**0.10**–**0.20**	0.16–**0.20**	**0.17**–0.19	**0.17**–**0.20**	0.16–0.17
AntVIpt [mm]	1.03–1.24	**1.01**–**1.27**	1.18–1.32	**1.11**–**1.33**	1.13–1.25
AntVIpt / AntIII [times]	**1.3**–1.4	1.5–**1.8**	**1.5**–1.7	1.7–**1.9**	1.7–1.9
AntVIpt / AntVIb [times]	**5.4**–6.9	5.5–**7.1** [~ 6.0]	6.6–7.1	**6.4**–**7.2** [5.8–7.2]	6.6–7.8
Femur of hind legs [mm]	0.97–**1.38**	**0.87**–1.23	1.05–**1.20**	**0.88**–1.10	0.37–0.38
Tibia of hind legs [mm]	1.85–**2.50**	**1.70**–2.25	2.05–**2.38**	**1.85**–2.27	0.73–0.74
Ultimate rostral seg. [mm]	0.16–**0.18**	**0.15**–**0.18** [0.16]	0.16–**0.17**	**0.15**–**0.17**	0.15
Ultimate rostral segment / its basal width [times]	2.2	**2.1**–**2.6**		2.6–2.8	
Ultimate rostral seg. / AntVIb [times]	0.9–**1.6**	**0.8**–1.1	0.9–**1.0**	**0.8**–0.9	0.9
Ultimate rostral seg / 2^nd^ seg. hind tarsi [times]	1.2–**1.4**	**1.1**–**1.4** [1.3]	**1.2**–1.4	1.3–**1.5** [**1.2**–1.3]	1.4
2^nd^ seg. hind tarsi [mm]	**0.11**–**0.15**	0.12–0.14	0.12–**0.14**	**0.11**–0.13	0.11
Siphunculus [mm]	0.48–0.61	**0.44**–**0.65**	0.44–0.53	**0.41**–**0.54**	0.39
*SPW* [mm]	0.05–**0.07**	**0.04**–**0.07**	0.04–0.5	0.04–0.05	0.04
*SSW* [mm]	0.07–**0.11**	**0.06**–0.10	**0.07**–0.08	**0.07**–**0.09**	0.06
Siphunculus / body [mm]	**0.17**–0.21	0.19–**0.23**	**0.16**–0.18	0.17–**0.20**	0.18
Siphunculus / AntIII [times]	**0.7**–0.8	**0.7**–**0.9**	**0.6**–0.7	**0.6**–**0.8**	0.6
Siphunculus / *SPW* [times]	**8.1**–9.7	8.5–**11.2**	**9.0**–11.0	9.2–**13.5**	11.3
Siphunculus / S*SW* [times]	**5.1**–6.9	5.6–**7.9**	6.0–6.6	**5.0**–**7.2**	6.6
*SSW* / *SPW* [times]	1.3–**1.8**	**1.2**–**1.8**	**1.4**–1.8	**1.4**–**2.1**	1.7
Cauda [mm]	0.24–0.30	**0.20**–**0.31**	0.21–**0.25**	**0.19**–**0.25**	0.15
Cauda / siphunculus [times]	0.5	**0.4**–**0.5** [0.5]	0.5	**0.4**–**0.5** [0.4–0.5]	0.4
Cauda / its basal width [times]	**1.3**–1.4	**1.3**–**1.8**	**1.2**–1.3	**1.2**–**1.4**	1
**Secondary sensoria** on…					
… AntIII [quantity]	0	0 [0]	14–**19**	**4**–**19** [6–17]	58–61
… AntIV [quantity]	0	0 [0]	0 [0]	0 [0]	15–33
… AntV [quantity]	0	0 [0]	0 [0]	0 [0]	7–12
**Setae** on…					
… head, dorsal med. [μm]	25–**30**	**17**–**30**	**16**–25	17–**28**	23
… head, dorsal med. / *D* [times]	0.7	**0.5**–**1.2**	**0.4**–0.7	0.5–**0.8**	0.7
… AntIII [μm]	15–**18**	**10**–**18**	15–18	**10**–**20**	18
… AntIII / *D* [times]	0.4–0.5	**0.3**–**0.6**	0.4–0.5	**0.3**–**0.6**	0.5
… penultimate rostral seg. [quantity]	20–**29**	**18**–24	20–**23**	**16**–19	18
… ultimate rostral seg., accessory [quantity]	**7**–11	**7**–**12** [9]	10–11	**9**–**14** [9]	11
… ultimate rostral seg., accessory [μm]	35–**48**	**25–3**5	**28**–35	30–**40**	
… hind femur, dorsal [μm]	**10**–**20**	**10**–13	**17**–**25**	**17**–23	
… hind femur, dorsal / *D* [times]	**0.3**–0.5	**0.3**–**0.6**	0.6–**0.8**	**0.5**–0.7	
… hind tibia, dorsal medial [μm]	22–**25**	**15**–23	20–**23**	**15**–20	
… hind tibia, dorsal medial / *D* [times]	**0.5**–0.7	**0.5**–**1.0**	0.6–**0.7**	**0.5**–**0.7**	
… Abd.2-Abd.5, spinal per segment [quantity]	10–12	**6**–**15**	10–14	**9**–**15**	
… Abd.2-Abd.5, spinal [μm]	10–14	**8**–**20**	**10**	**10**–**18**	13
… Abd.2-Abd.5, spinal / *D* [times]	0.3–0.4	**0.2**–**0.6**	**0.3**	**0.3**–**0.5**	0.4
… Abd.2-Abd.5, ventral [μm]	**20**–35	25–**38**	**23**–**35**	25–**35**	20
… Abd.2-Abd.5, ventral / *D* [times]	**0.5**–1.0	0.8–**1.7**	**0.7**–**1.1**	0.8–**1.1**	0.6
… Abd.8 [quantity]	5–**8**	**4**–**8** [6]	6–**8**	**4**–**8** [5–7]	4
… Abd.8 [μm]	**25**–**53**	30–40	30–**38**	**27–3**5	48
… Abd.8 / *D* [times]	**0.7**–1.5	0.9–**1.7**	**0.**9–1.0	**0.8**–**1.1**	1.5
… genital plate, discal [quantity]	**2**–**5**	**2–5**	**2**–3	**2–4**	///
… genital plate, posterior [quantity]	**12**–16	16–**24**	**14–1**5	17–**24**	///
… cauda [quantity]	**7**	**3**–6 [5]		4–7 [5]	5

Additionally several qualitative features, particularly the absence of cilia in the relatively thick edge of the primary sensoria, allow us to separate this species from species in other genera of Macrosiphini with similar characteristics, establishing a new genus, which is named *Delfinoia*.

### Delfinoia

Taxon classificationAnimaliaHemipteraAphididae

Nieto Nafría & Mier Durante gen. n.

http://zoobank.org/94ECE792-62B4-4CBD-BAB4-8DB36BEDB5C0

Figs 2 , 3 , 4

#### Diagnosis.

Aphid genus belongig to tribe Macrosiphini (Aphididae, Aphidinae) with primary sensoria on antennal segments V and VI with thick and non-ciliated edge (Fig. 4).

#### Description.

Macrosiphine aphid with (1) primary sensoria with thick and non-ciliated edge (Fig. 4), in addition to presence of (2) moderately divergent and smooth frontolateral tubercles and a small frontomedial tubercle and small frontal sinus in apterous viviparous females (Figs 2, 3A), (3) long antenna with long antennal segment VI terminal process (Fig. 2), (4) long and moderately swollen siphunculi (Figs 2, 3E), and also (5) secondary sensoria on antennae absent in apterous viviparous females and present on segment III in alate viviparous females (Figs 2, 3G), (6) penultimate and ultimate rostral segments provided with many robust, long and pointed setae (Fig. 3C), (7) first segments of tarsi with three setae, (8) dorsum scarcely sclerotized, mainly unpigmented in wingless forms and with marginal and intersegmental sclerites in winged forms (Fig. 2), (9) rugose and pale spiracular sclerites and circular or subcircular spiracular apertures (Fig. 3D), (10) truncated and short setae on most part of body dorsum and appendages (Fig. 3B), (11) siphunculi tenuously ornamented in the proximal part, smooth on remaining length, and provided with few and not always complete lines under the flange (Figs 2, 3E), (12) triangular and relatively short cauda (Table 1, Figs 2, 3F), and (13) dusky bordered forewing cubital veins in winged forms (Fig. 2).

#### Type species.

*Amphorophora peruviana* Essig, 1953.

#### Taxonomic discussion.

Ten genera and one subgenus of Macrosiphini known in the Americas have more or less developed and divergent or parallel frontolateral tubercles, long antennae and elongate swollen siphunculi (characters 2, 3 and 4); they are *Amphorophora* Buckton, 1876, *Delphiniobium* Mordvilko, 1914, *Gibbomyzus* Nieto Nafría, Pérez Hidalgo, Martínez-Torres & Villalobos Muller, 2013, *Glabromyzus* Richards, 1960, *Hyperomyzus* Börner, 1933, *Illinoia* Wilson, 1910, *Rhopalomyzus* Mordvilko, 1921 and *Ucrimyzus* Mier Durante & Pérez Hidalgo, 2013, *Utamphorophora* and *Wahlgreniella*, and the subgenus Picturaphis Blanchard, 1922 which is currently included in genus *Microparsus* Patch, 1909.

Feature 1 is the most distinctive character of the new genus, and is very exceptional in Macrosiphini, and features 5 to 13 in combination help to separate the new genus from any of the above mentioned genera, although they are present in some of them.

The novelty of the genus could be assured with complete certainty by the analysis of some genetic marker, which cannot be carried out at present because all the known material of the species is mounted on microscopic slides.

#### Etymology.

The name *Delfinoia* is in honour of Dr. Miguel Ángel Delfino (retired professor of entomology, University of Cordoba (Argentina), aphidologist and good friend for decades), who was the author of *W. australis*.

### Delfinoia peruviana

Taxon classificationAnimaliaHemipteraAphididae

(Essig, 1953) comb. n.

Figs 2A, C , 3 , 4

Amphorophora peruviana Essig: Essig, 1953; Proceedings of the California Academy of Sciences, Fourth Series, 28 (3): 133 & 135.
Wahlgreniella australis Delfino: Delfino, 1981; Revista de la Sociedad Entomológica Argentina, 40 (1-4): 183–186; **syn. n.**
Utamphorophora peruviana (Essig, 1953): Eastop, 1997; in Remaudière (G.) & Remaudière (M.), Catalogue des Aphididae du monde / Catalogue of the World’s Aphididae (HomopteraAphidoidea): page 158.

#### Description.

**Apterous viviparous females** (redescription, from 30 studied specimens [see “Materials and methods”] and original descriptions of both nominal species).

Colour unknown when alive, possibly green or light green, and perhaps, from Essig’s drawing, with two small dark spots on each side of several abdominal segments, brown cauda and dark brown or blackish brown siphunculi. When mounted variably light yellow, with head, including antennae and rostrum, legs, siphunculi, anal plate and cauda more or less pigmented (see below). Quantitative characters are in Table 1. ***Head***. Brownish yellow. Frons sinuated, with broadly divergent and moderately developed frontolateral tubercles and low frontomedial tubercle. Dorsum smooth and ventrum with stretch marks. Setae of first and second dorsal row (each with two setae) and internal setae of third dorsal row (with four setae) similar in length to each other; external setae of third row approximately half as long as the other six. These eight dorsocephalic setae, the frontolateral apical setae and the three ventrolateral setae on each side (near the margins of the antennal alveoli) have truncate apices; other ventral setae, including those on clypeus and on mandibular and maxillar laminae, are pointed. Antennal segment I slightly pigmented and mostly smooth, with its inner side somewhat darker and gently scabrous; segment II also slightly pigmented, dorsally smooth and ventrally scabrous. Antennal segment III also pale, with a smoky apical ring, and tenuous cuticular ornamentation, which is more marked on the ventral face of its 1/5 proximal portion. Its subarticular constriction is less marked than in some other aphids; possibly the antennal flagellum has reduced mobility with respect to the pedicel as a result of this structural feature. Antennal segment IV softly imbricated and mostly pale, with smoky small proximal ring and distal portion; segment V similar to segment IV but more intensely imbricated and with a longer and more pigmented distal portion. Antennal segment VI brown and imbricated. Several setae on segment VI are pointed and longer than other antennal setae, which are similar in shape and size to dorsocephalic ones. Secondary sensoria absent. Primary sensoria on antennal segments V and VI with thick, sclerotic and non-ciliate margins. Satellite sensoria grouped ventrad to the primary sensorium. Rostrum extends back to slightly beyond hind coxae. Penultimate and ultimate rostral segments similar in length and colour (light brown) and bearing many robust, rigid and pointed setae. ***Thorax***. Paler than head and generally devoid of marked cuticular ornamentation. Spiracular sclerites rugose and unpigmented, spiracular apertures circular or subcircular. Marginal papillae on prothorax if present are small, flat and unpigmented. Both dorsal and ventral setae similar in shape and size to those on anterior abdominal segments. Tarsi and apex of tibiae pale brown, rest of legs brownish yellow. Setae on femora and most of those on tibiae short and with truncate apices; setae on coxae (which are longer than others), trochanters and tarsi pointed, as also are dorsoapical tibial setae. First segments of tarsi with three setae. ***Abdomen***. In general paler than head. Spiracular sclerites and apertures similar to those on thorax. Intersegmental sclerites inconspicuous. Small presiphuncular sclerites small, postsiphuncular sclerites relatively wide, and a narrow transverse stripe on segment VIII; all of these sclerites spinuled and pale yellow. Dorsal setae short and with truncate apices, except those on abdominal segment VIII, which are pointed. Ventral setae pointed. One specimen (paratype) has one marginal tubercle on abdominal segment IV, small and pale. Siphunculi light brown, swollen over distal half of length, smooth or nearly smooth for most of length, and with three or four complete or incomplete circular lines below the flange, which is protruding and relatively thick. Genital plate very pale; anal plate with similar pigmentation to cauda, which is triangular with blunt apex. Setae on these plates and cauda pointed.

**Alate viviparous females** (redescription, from 12 studied specimens [see “Materials and methods” section] and from original descriptions of both nominal species). Fig. 2B, D.

Colour unknown when alive, possibly with dark brown or black head and thorax, including antennae and legs, and green abdomen with dark brown lateral spots, cauda and siphunculi. Quantitative characters are in Table 1; qualitative characters like those of apterae are not mentioned. ***Head***. Brown with darker areola around each ocellus. Dorsum with tenuous ornamentation. Frontolateral tubercles very low and frontomedial tubercle inconspicuous. Antennae homogeneously brown. Antennal segment III with secondary sensoria, which are similar in shape to the primary ones and variable in size, more-or-less aligned over almost the entire length. ***Thorax***. Legs brown, with paler coxae, trochanters and proximal part of femora. Fore wings with veins well-marked and the cubital veins dark-bordered; hind wings veins also well-marked but not bordered. ***Abdomen***. Pale in general. Sclerites variably pigmented, sometimes as pale as the rest of the abdominal cuticle. Intersegmental sclerites smooth. Marginal sclerites on segments I - VII spinuled, the postsiphuncular sclerites being wider than the others. Setiferous spinal and pleural sclerites present on segment VIII and sometimes on segments VI and VII, all of them spinuled and usually pale or very pale. One specimen (holotype) has four small, ill-defined and pale marginal tubercles on abdominal segments II, III (on both sides) and IV; another specimen (paratype) also has similar tubercles on one side of abdominal segments III and IV.

**Males** (from one specimen, see “Materials and methods” section). Fig. 2E–F. Winged; similar to alate viviparous females in general aspect, pattern of sclerotisation, extent of pigmentation and cuticular ornamentation. Colour when alive unknown. Secondary sensoria present on antennal segments III, IV and V. Hind wings have a single oblique vein, which could well be an anomaly, although the presence of an identical anomaly in both wings is strange. Two small pale abdominal marginal tubercles present. Parameres broad, curved back, very dark brown and provided with many, rigid, pointed and relatively long hairs. Quantitative characters are in Table 1.

#### Biology.

It is certain that *Delfinoia peruviana* feeds on plants of one or more species of *Cayaponia* in Argentina, and perhaps also in Peru (see “Introduction”). The species has been also caught on a cruciferous plant in Cordoba (Argentina). The collector of those specimens, L. Bahamondes, was an experienced (but now deceased) entomologist and a connoisseur of Argentinean flora, so one can be certain that the specimens were collected on a plant of family Brassicaceae, but it is also conceivable that the specimens collected had fallen from some vine of the genus *Cayaponia*.

#### Distribution.

The species is currently known in two localities (one in Peru and the other in Argentina) that are 2,200 kilometers distant from each other. Possibly the species can be found in much of northern Argentina, southern Peru and also in eastern Bolivia and southwestern Brazil.

## Supplementary Material

XML Treatment for Delfinoia

XML Treatment for Delfinoia peruviana
